# Supplementary material for: Bovine Polyomavirus-1 (Epsilonpolyomavirus bovis): An Emerging Fetal Pathogen of Cattle That Causes Renal Lesions Resembling Polyomavirus-Associated Nephropathy of Humans
Source: Viruses. 2022 Sep 14;14(9):2042. doi: 10.3390/v14092042 (PMC9502773; doi:10.3390/v14092042)
Supplement: Supplementary file 1 [file viruses-14-02042-s001.zip › viruses-1889117-supplementary.pdf]

viruses

Article

Bovine Polyomavirus-1 (*Epsilonpolyomavirus bovis*): An Emerging Fetal Pathogen of Cattle that Causes Renal Lesions Resembling Polyomavirus-associated Nephropathy of Humans

Federico Giannitti [1,*], Caroline da Silva Silveira [1], Hannah Bullock [2], Marina Berón [1], Sofía Fernández-Ciganda [1], María José Benítez-Galeano [3], Nélida Rodríguez-Osorio [3], Luciana Silva-Flannery [4], Yisell Perdomo [1], Andrés Cabrera [5,6], Rodrigo Puentes [5], Rodney Colina [7], Jana M. Ritter [4] and Matías Castells [7,*]

[1] Plataforma de Investigación en Salud Animal, Instituto Nacional de Investigación Agropecuaria (INIA), Estación Experimental La Estanzuela, Colonia 70006, Uruguay
[2] Synergy America Inc., Atlanta, GA 30329, USA
[3] Unidad de Genómica y Bioinformática, Departamento de Ciencias Biológicas, Centro Universitario Regional (CENUR) Litoral Norte, Universidad de la República, Salto 50000, Uruguay
[4] Infectious Diseases Pathology Branch, Centers for Disease Control and Prevention (CDC), Atlanta, GA 30329, USA
[5] Facultad de Veterinaria, Universidad de la República, Montevideo 13000, Uruguay
[6] Laboratorio de Interacciones Hospedero-Patógeno, Institut Pasteur de Montevideo, Montevideo 11400, Uruguay
[7] Laboratorio de Virología Molecular, Departamento de Ciencias Biológicas, Centro Universitario Regional (CENUR) Litoral Norte, Universidad de la República (UDELAR), Salto 50000, Uruguay
* Correspondence: fgiannitti@inia.org.uy (F.G.) and matiascastellsbauer@gmail.com (M.C.)

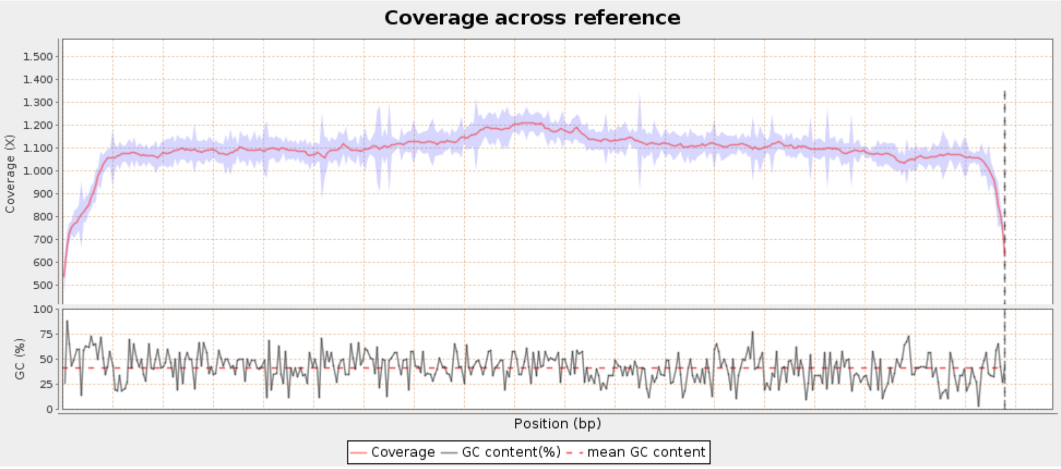

Figure S1: Coverage of the Oxford Nanopore Technology reads across the genome. In the upper panel the coverage of the reads across the reference genome (GenBank accession number D13942) is shown. In the lower panel, the GC content (%) and the mean GC content across the reference genome is shown.

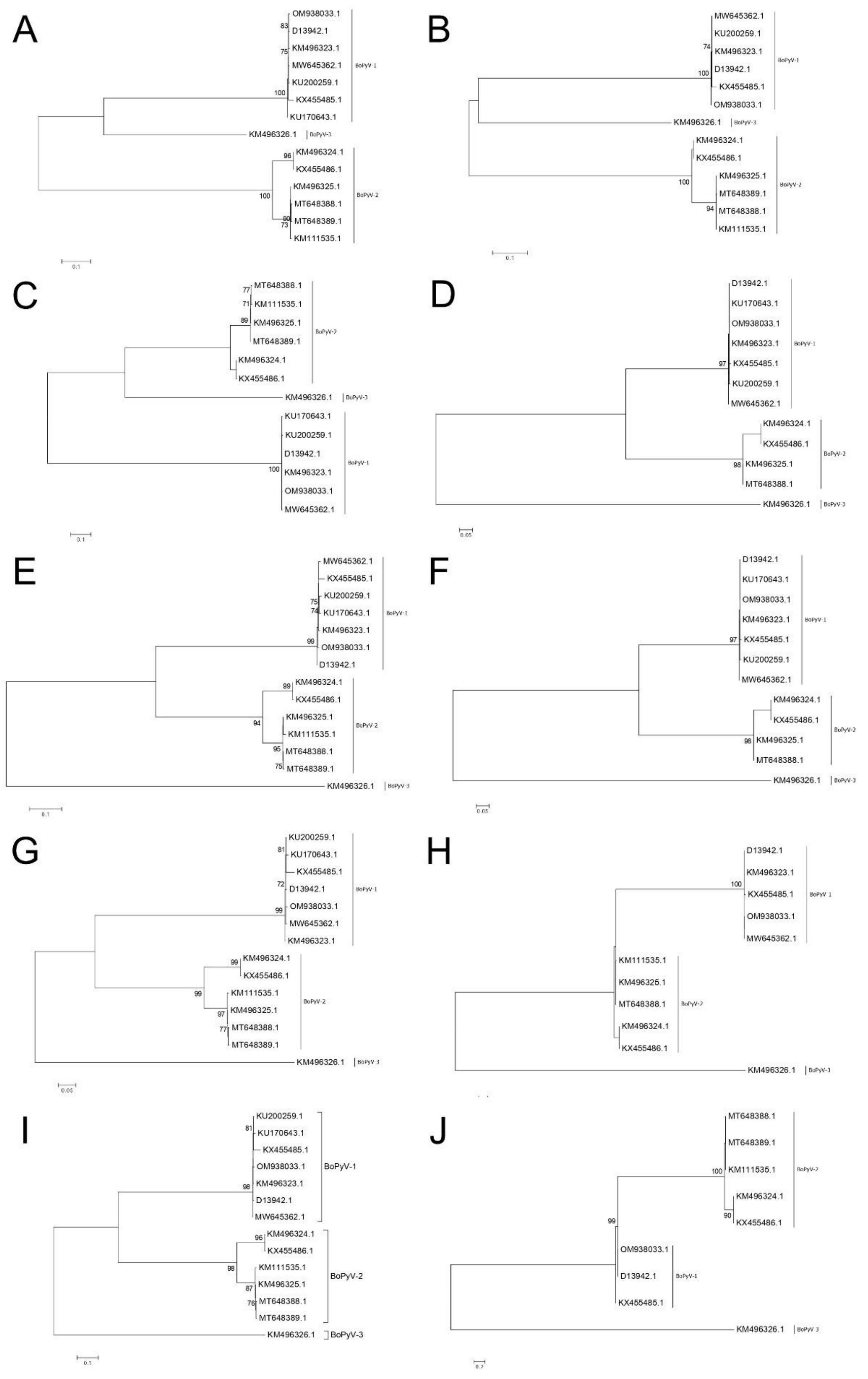

Figure S2: Phylogenetic analyses of LT antigen, ST antigen, VP1, VP2 and VP3. The maximum likelihood trees obtained with IQ-TREE web server with nucleotide (A, C, E, G, and I) and amino acid (B, D, F, H, and J) sequences are shown. All the BoPyV sequences available in the database were downloaded and together with BoPyV-1/Faber/2021/Uy were aligned with Clustal W. The best substitution model was jointly obtained with the tree using the IQ-TREE web server. The

analyses were performed using LT antigen (A and B), ST antigen (C and D), VP1 (E and F), VP2 (G and H), and VP3 (I and J). Some sequences are not shown in the trees because IQ-TREE discarded them since they were identical to others. Branch support analysis was SH-aLRT branch test implemented in the IQ-TREE web server.
